# A new class A beta-lactamase gene *bla*_CAE-1_ coexists with *bla*_AFM-1_ in a novel untypable plasmid in *Comamonas aquatica*

**DOI:** 10.1038/s41598-023-28312-w

**Published:** 2023-03-03

**Authors:** Ying Li, Chengju Fang, Xu Wang, Qian Liu, Yichuan Qiu, Xiaoyi Dai, Luhua Zhang

**Affiliations:** 1 The School of Basic Medical Science and Public Center of Experimental Technology, Southwest Medical University, Luzhou, 646000 Sichuan Province China; 2 Immune Mechanism and Therapy of Major Diseases of Luzhou Key Laboratory, School of Basic Medical Science, Southwest Medical University, Luzhou, 646000 Sichuan Province China; 3 Department of Clinical Laboratory, The Affiliated Traditional Chinese Medicine Hospital, Southwest Medical University, Luzhou, Sichuan China

**Keywords:** Microbiology, Antimicrobial resistance

## Abstract

Antimicrobial resistance, especially carbapenem resistance, poses a serious threat to global public health. Here, a carbapenem-resistant *Comamonas* *aquatica* isolate SCLZS63 was recovered from hospital sewage. Whole-genome sequencing showed that SCLZS63 has a 4,048,791-bp circular chromosome and three plasmids. The carbapenemase gene *bla*_AFM-1_ is located on the 143,067-bp untypable plasmid p1_SCLZS63, which is a novel type of plasmid with two multidrug-resistant (MDR) regions. Notably, a novel class A serine β-lactamase gene, *bla*_CAE-1_, coexists with *bla*_AFM-1_ in the mosaic MDR2 region. Cloning assay showed that CAE-1 confers resistance to ampicillin, piperacillin, cefazolin, cefuroxime, and ceftriaxone, and elevates the MIC of ampicillin-sulbactam two-fold in *Escherichia coli* DH5α, suggesting that CAE-1 functions as a broad-spectrum β-lactamase. Amino acid sequences analysis suggested that *bla*_CAE-1_ may originate from *Comamonadaceae.* The *bla*_AFM-1_ in p1_SCLZS63 is located in a conserved structure of IS*CR29*-Δ*groL*-*bla*_AFM-1_-*ble*-Δ*trpF*-ΔIS*CR27*-*msrB*-*msrA*-*yfcG*-*corA*. Comprehensive analysis of the *bla*_AFM_-bearing sequences revealed important roles of IS*CR29* and ΔIS*CR27* in the mobilization and truncation of the core module of *bla*_AFM_ alleles, respectively. The diverse passenger contents of class 1 integrons flanking the *bla*_AFM_ core module make the complexity of genetic contexts for *bla*_AFM_. In conclusion, this study reveals that *Comamonas* may act as an important reservoir for antibiotics-resistance genes and plasmids in the environment. Continuous monitoring for the environmental emergence of antimicrobial-resistant bacteria is needed to control the spread of antimicrobial resistance.

## Introduction

*Comamonas* spp. are a group of Gram-negative, nonfermenting and rod-shaped bacteria belonging to the *Comamonadaceae* family of the phylum *Proteobacteria*^1^. This organism frequently grows in a wide range of habitats, such as wastewater, wetlands, soil, and hospital environments^2^, and has been reported as one of the major members of microbial communities in wastewater bioaugmentation and bioremediation^3^. In spite of its uncommon pathogenesis, some *Comamonas* species have been increasingly reported to be closely associated with invasive infections in humans, such as bacteremia^4,5^, urinary tract infection^6^, intra-abdominal infection^7^, and meningitis^8^.

β-lactams are the most commonly used antibiotics in clinical settings due to their safety, efficacy, and broad-spectrum of activity^9^. The biggest challenge to the use of β-lactams is the production of β-lactamases, which is the most common and important resistance mechanism for β-lactamases in Gram-negative bacteria^10^. Based on the amino acid sequence identity, β-lactamases are divided into four molecular classes (A-D)^11^. Classes A, C, and D β-lactamases hydrolyze their substrates through the formation of an acyl enzyme with an active-site serine, while the hydrolytic reaction of class B β-lactamases requires one or two essential zinc ions^11^. At present, the widespread of extended-spectrum β-lactamase (ESBL)- and carbapenemase-producing organisms pose a serious challenge for clinical management and public health for their hydrolytic activity against expanded-spectrum of β-lactam antibiotics^9,12,13^. Hospital wastewater is a complex matrix containing a high abundance of bacteria combined with sublethal antibiotic concentrations from clinical settings, which serves as a hot spot for the evolution and dissemination of antimicrobial resistance (AMR), and also a reservoir of novel resistance genes^14^. Marathe et al.^15^ demonstrated that hospital sewage effluent creates a niche where pathogens acquire novel antibiotic resistance genes (ARGs), including carbapenemases genes. Hem et al.^16^ isolated a subset of carbapenem-resistant *Comamonas* strains from wastewater, of which most possess novel unknown resistance mechanisms for carbapenems. Therefore, wastewater is an ideal place to identify novel ARGs.

To date, carbapenem resistance genes *bla*_NDM_^17^, *bla*_IMP-8_^18^, and *bla*_GES-5_^16^ have been reported in *Comamonas* isolates. AFM-1 is a newly emergent carbapenemase that was first identified in a clinical *Alcaligenes faecalis* strain AN-70 in China and was later reported in *Pseudomonas aeruginosa*^19^ and *Aeromonas hydrophila*^20^, in which IS*CR* elements are involved in the mobilization of *bla*_AFM-1_. In silico analysis with the GenBank database indicated four variants of AFM (AFM-1 to -4) that were also present in *Stenotrophomonas maltophilia, Bordetella trematum,* as well as *Comamonas* isolates. In this study, we described a novel type of multidrug-resistance plasmid carrying the carbapenemase gene *bla*_AFM-1_ in a *Comamonas aquatica* strain from hospital sewage. Of note, we also characterized a novel class A serine β-lactamase gene, *bla*_CAE-1_, which coexists with *bla*_AFM-1_ on the plasmid. In addition, we performed a comprehensive genomic comparison of *bla*_AFM_-bearing sequences to gain a better understanding of the dissemination patterns of this novel carbapenemase gene.

## Materials and methods

### Bacterial isolation and in vitro susceptibility testing

*C. aquatica* SCLZS63 was recovered from the sewage outlet of the affiliated hospital of Southwest Medical University, Sichuan Province, China, in November 2019. As previously described^21^, 5 ml of water sample was concentrated by centrifugation for 5 min at 5000 g, and the sediment was resuspended in sterile 0.9% NaCl solution, then, the bacterial suspension was plated onto MacConkey agar containing meropenem (2 μg/ml) and incubated for 24 h at 37 °C. A single colony was picked, and initial species identification was performed by sequence analysis of 16S rRNA gene after PCR amplifying and Sanger sequencing^22^. The susceptibility to ceftazidime, cefotaxime, aztreonam, meropenem, and imipenem for SCLZS63 was examined by using the broth microdilution method and interpreted according to the Clinical and Laboratory Standards Institute (CLSI) guidelines for other non-enterobacterales bacteria^23^.

### Genome sequencing and analysis

Genomic DNA of SCLZS63 was obtained using the QIAamp DNA Mini Kit (Qiagen), and the purified DNA was subjected to whole-genome sequencing on a HiSeq 2000 platform (Illumina, San Diego, CA, USA) using a paired-end library with an insert size of 150 bp, followed by the long-read MinION Sequencer (Nanopore, Oxford, UK). The de novo hybrid assembly of the Illumina reads and MinION reads were carried out by using Unicycler v0.4.3^24^. Gene annotation for the assembled genomes was performed with the RAST tools^25^ and BLASTp/BLASTn searches against the UniProtKB/SwissProt database^26^. Bacterial precise species were identified using pairwise ANI analysis between strain SCLZS63 and reference genomes of *Comamonas* with the online software JSpeciesWS (https://jspecies.ribohost.com/jspeciesws/). A > 96% ANI cut-off was used for species circumscription^27^. Plasmid incompatibility types, antibiotic resistance genes, and insertion elements were predicted using PlasmidFinder 2.1 (95%, minimum threshold for identity; 60%, minimum coverage)^28^, ResFinder (90%, minimum threshold for identity; 60%, minimum coverage)^29^, and ISfinder^30^.

### Phylogenetic analysis

Amino acid sequences of 17 class A β-lactamases were retrieved from the Beta-Lactamase DataBase (BLDB, http://bldb.eu/), and were utilized for the phylogenetic analysis. Sequences were aligned using the program Clustal W^31^. A maximum-likelihood tree was generated by MEGA 6 software^32^, with 1000 bootstrap replicates, and was then annotated using iTOL^33^.

### Gene cloning

PCR amplification of the complete coding sequence of *bla*_CAE-1_ and its promoter region from *C. aquatica* SCLZS63 was performed using the primers *bla*_CAE_-F: 5′-cagcaaatgggtcgcggatccGCTTACTTTCACTCATGACGTCACC-3′and *bla*_CAE_-R: 5′-gtggtggtggtggtgctcgagGGATGTTGGAAGACCCGACC-3′. The resulting PCR fragment was then ligated into an expression vector pET28a using a ClonExpress® II One Step Cloning Kit (Vazyme, China) to construct pET28a-*bla*_CAE-1_, which was introduced into *Escherichia coli* DH5α by chemical transformation. Potential transformants containing the recombinant plasmid were selected on Luria–Bertani (LB) agar plates containing 50 mg/L kanamycin and verified by PCR assays. The susceptibility to antimicrobial agents (ampicillin, piperacillin/tazobactam, ampicillin/sulbactam, cefazolin, ceftriaxone, ceftazidime, cefotaxime, cefepime, aztreonam, ertapenem, meropenem, and imipenem) for the recombination strain was performed using the broth microdilution method according to the CLSI guidelines with *E. coli* strain ATCC 25922 as the quality control strain. *E. coli* DH5α containing the empty pET28a served as a negative control.

### Conjugation and electroporation experiments

Conjugation experiments were performed using both broth- and filter-based methods, with the azide-resistant *E. coli* J53 as the recipient, as described previously^34^. Equal amounts of donor and recipient cells at the exponential stage (the optical density at 600 nm reaches ~ 0.5) were mixed and incubated at 37 °C in LB broth or on the filter that was placed on an LB agar plate overnight. Subsequently, cells were resuspended and diluted in 0.9% NaCl, and potential transconjugants were selected on LB agar plates containing 150 µg/ml sodium azide and 4 µg/ml cefotaxime. Conjugation assays were repeated with different donor/recipient ratios.

Electroporation was carried out with *E. coli* DH5α as the recipient. Plasmids of *C. aquatica* SCLZS63 were extracted using the E.Z.N.A. plasmid Mini Kit I (OMEGA, Bio-Tek, USA), verified by agarose gel electrophoresis, and then transferred by electroporation (Micro-Pulser electroporator; Bio-Rad, USA) into DH5α competent cells. Transformants were selected on LB agar plates containing 4 µg/ml cefotaxime. The presence of *bla*_CAE-1_ in the transformant was examined by PCR assays with primers *bla*_CAE_-F/R.

### Comparative analysis of ***bla***_AFM_-bearing sequences

To obtain the *bla*_AFM_-bearing sequences, a BLASTn with standard options was performed with the nucleotide sequences of *bla*_AFM-1_ (GenBank accession no. NG_063835) as a query in the NCBI GenBank database. Plasmids and chromosomes with a full-length hit to *bla*_AFM-1_ (100% query coverage and ≥ 99.88% identity) were selected. Alignments of the *bla*_AFM_-bearing sequence were performed using BLASTn and visualized with Easyfig v 2.2.3.

## Results

### Genome feature of *C. aquatica* strain SCLZS63

Strain SCLZS63 was initially identified as a *Comamonas* strain, which was resistant to ceftazidime (MIC, 128 μg/ml), cefotaxime (MIC, 128 μg/ml), aztreonam (MIC, 256 μg/ml), meropenem (MIC, 16 μg/ml), and imipenem (MIC, 16 μg/ml), respectively. Whole-genome sequencing (WGS) revealed that SCLZS63 contains one single circular chromosome with a size of 4,048,791 bp and an average GC content of 64.53%, and three circular plasmids, p1_SCLZS63, p2_SCLZS63, and p3_SCLZS63 (Table 1). SCLZS63 belongs to *C. aquatica* as it had 96.55% identity (81.16% coverage) to the *C. aquatica* reference strain NEB418 by average nucleotide identity (ANI) analysis. It had 9 known ARGs, mediating resistance to aminoglycosides [*aac(6')-IIa*, *aph(6)-Id*, and *aph(3'')-Ib*], β-lactams (*bla*_AFM-1_), macrolides [*mph*(E) and *msr*(E)], sulfonamides (*sul1*), trimethoprim (*dfrA5*), and amphenicols (*cmx*). Among them, *aph(6)-Id* and *aph(3'')-Ib* are located on the chromosome, while the remainder ARGs are carried by p1_SCLZS63.

**Table 1:** Summary of the genetic features of *C. aquatica* SCLZS63.

Chromosome/pasmid	Length (bp)	GC%	No. of predicted ORFs	Drug resistance genes
Chromosome	4,048,791	64.53	3852	*aph(6)-Id*, *aph(3'')-Ib*
p1_SCLZS63	143,067	56.68	141	*aac(6')-IIa*, *bla*_AFM-1_, *bla*_CAE-1_, *mph*(E), *msr*(E), *sul1*, *dfrA5*, *cmx*
p2_SCLZS63	9512	55.87	12	
p3_SCLZS63	1582	60.11	2	

–, not available.

### CAE-1 confers resistance to several β-lactams

**Figure 1:**
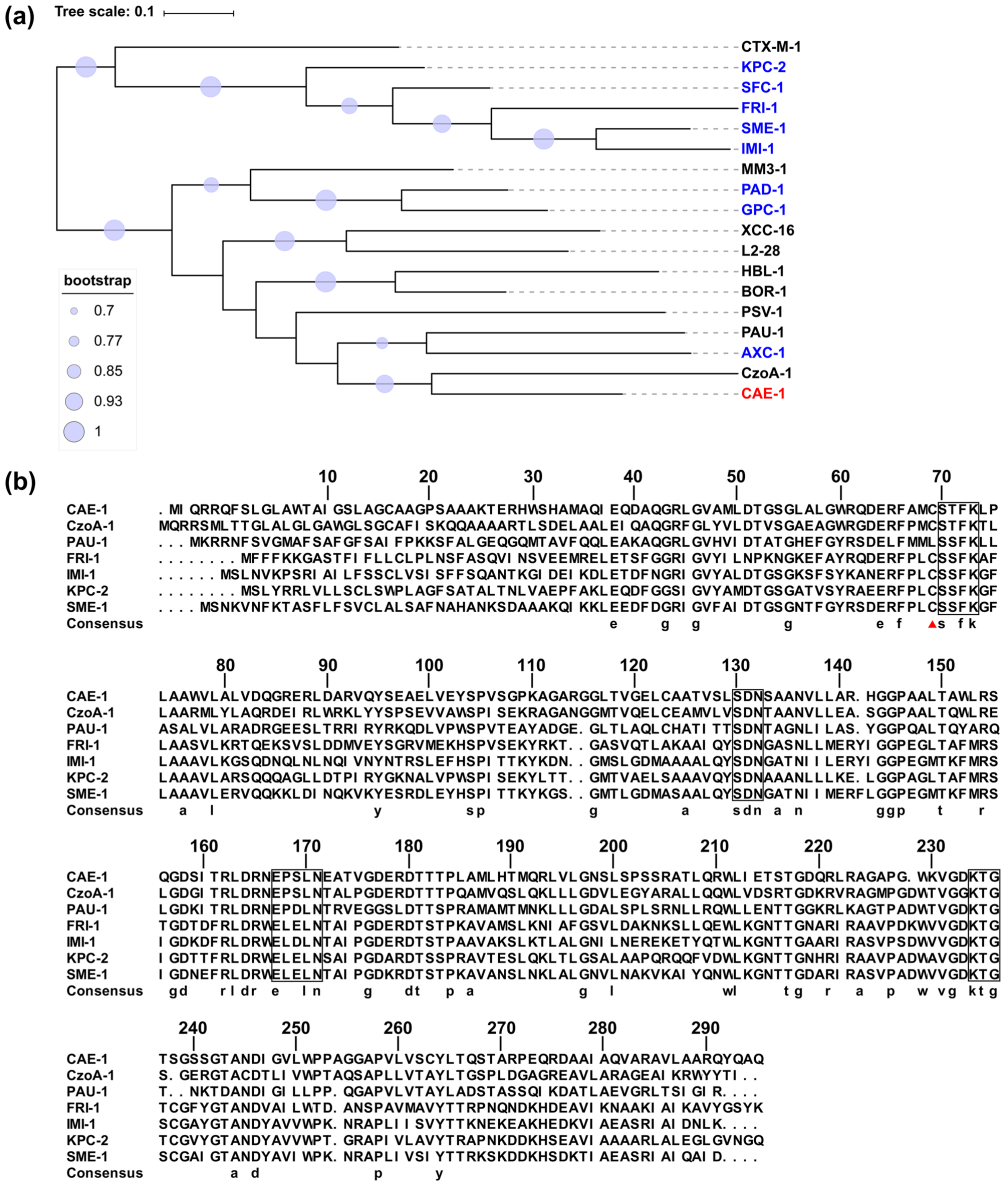
Comparison of CAE-1 with other known class A beta-lactamase. (**a**) An unrooted phylogenetic tree of CAE-1 and its close homologs and representative class A beta-lactamase. Proteins with carbapenemase activity are highlighted in blue and CAE-1 is in red. The tree scale indicates substitutions per site. The β-lactamases (GenBank accession numbers) are CTX-M-1(DQ915955), KPC-2 (LDDY01000008), SFC-1(AY354402), FRI-1(KT192551), SME-1 (Z28968), IMI-1 (U50278), MM3-1 (MK831000), PAD-1(LNTU01000040), GPC-1 (MH211206), XCC-16 (QUZR01000001), L2-28 (CP011010), HBL-1 (CP012077), BOR-1 (BX640443), PSV-1 (KU926347), PAU-1 (KU881625), AXC-1 (MF767301), and CzoA-1 (ADVQ01000062). (**b**) Amino acid sequence alignment of CAE-1 with CzoA-1, PAU-1, FRI-1, IMI-1, KPC-2 and SME-1. The residue numbers are positioned above the sequences according to the standard numbering scheme for the class A beta-lactamases^39^. The four conserved motifs of class A β-lactamases are outlined in black and the red triangle indicates the conserved residue ^69^C among class A carbapenemases.

Notably, a 909-bp open reading frame (ORF) encoding a putative 302-amino-acid class A β-lactamase was identified on p1_SCLZS63. The novel class A β-lactamase is most closely related to CzoA-1^35^ (Accession no. EFI60385), with only 52.7% amino acid identity, followed by PAU-1^36^ (48.8%, APC57487), and AXC-1 (48.3%, ATG32091) (Fig. 1A). Therefore, it was given a new family name CAE-1 (for *C. aquatica* enzyme). The novel CAE-1 contains the four conserved motifs of class A β-lactamases, namely, ^70^SXXK^73^, ^130^SDN^132^, ^166^EXXXN^170^, and ^234^KTG^236^^37,38^ (Fig. 1B). Only one amino acid residue ^69^C that is associated with carbapenemase activity was identified in CAE-1^37^ (Fig. 1B).

To determine whether CAE-1 mediates resistance to β-lactams or not, *bla*_CAE-1_ was cloned into the pET28a vector and transformed into *E. coli* DH5α. Compared with the control strain DH5α carrying the empty pET28a, the recombinant strain DH5α/pET28a-*bla*_CAE-1_ exhibited resistance to some β-lactams tested, including ampicillin, piperacillin, cefazolin, cefuroxime, and ceftriaxone (Table 2), and the MIC for ampicillin-sulbactam increased for two-fold. The acquisition of *bla*_CAE-1_ had no effect on the MICs of carbapenems and aztreonam. This finding implies that CAE-1 has broad-spectrum activity profiles, and functions as broad-spectrum β-lactamase.

**Table 2:** MICs for recombinant strain producing CAE-1 and control strains.

Antibiotic	MIC (μg/ml)		
	DH5α/pET28a-*bla*_CAE-1_	DH5α/pET28a	*E. coli* ATCC25922
Ampicillin	**≥ 32**	≤ 8	4
Piperacillin	**≥ 128**	**≤ **4	**≤ **4
Ampicillin/sulbactam	16/8	≤ 8/4	**≤ **2
Cefazolin	**≥ 64**	**≤ **2	**≤ **2
Cefuroxime	**≥ 64**	4	4
Ceftriaxone	**≥ 64**	**≤ **1	**≤ **1
Ceftazidime	**≤ **1	**≤ **1	**≤ **1
Cefotaxime	**≤ **1	**≤ **1	**≤ **1
Cefepime	**≤ **2	**≤ **2	**≤ **1
Aztreonam	**≤ **4	**≤ **4	**≤ **1
Ertapenem	**≤ **1	**≤ **1	**≤ **0.5
Meropenem	**≤ **1	**≤ **1	**≤ **0.25
Imipenem	**≤ **1	**≤ **1	**≤ **0.25

Resistant MIC′s are highlighted in bold.

### The mobilization and possible origin of ***bla***_CAE-1_

**Figure 2:**
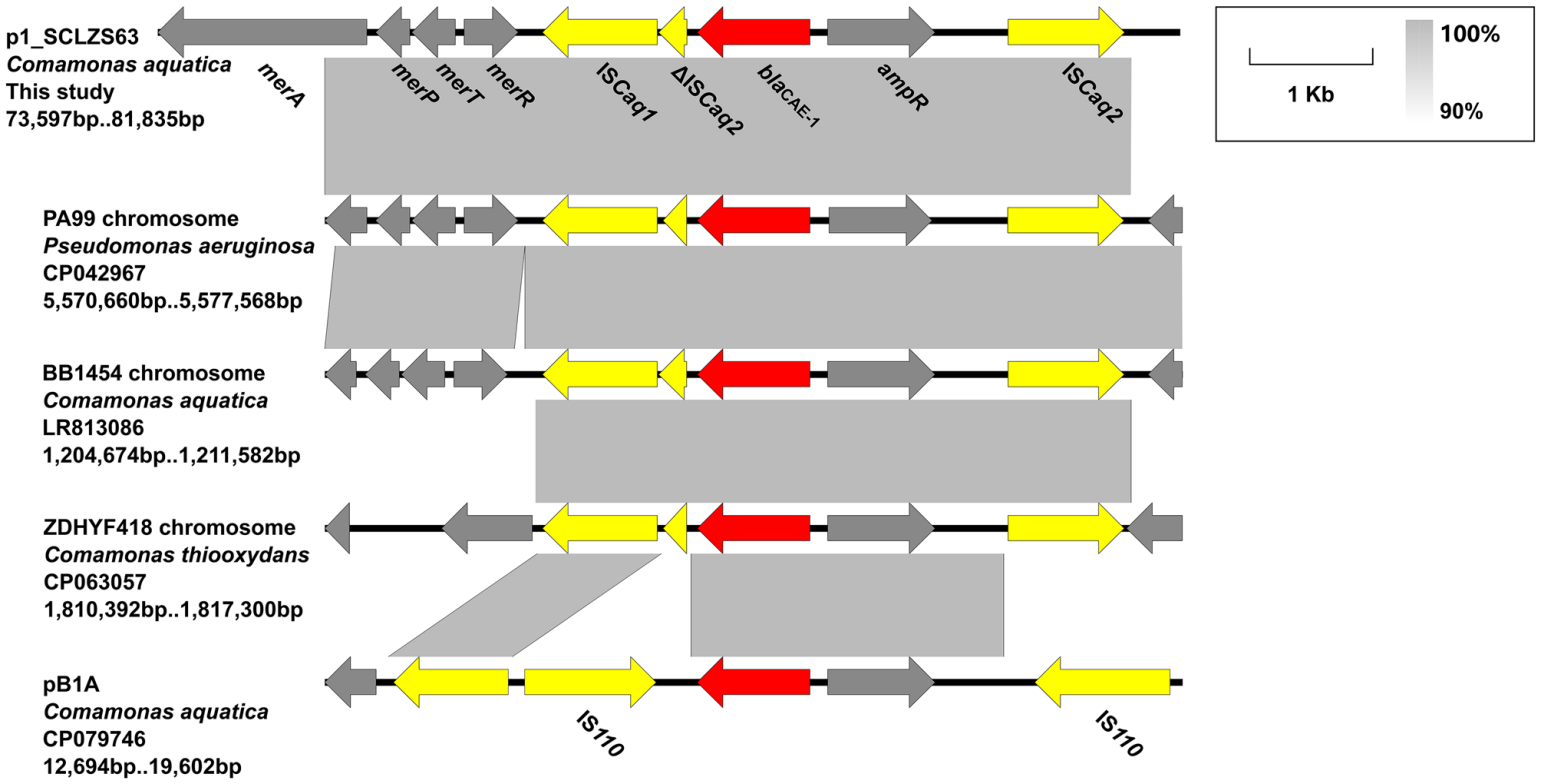
Genetic contexts of *bla*_CAE-1_. The position and transcriptional direction of ORFs are indicated by arrows. *bla*_CAE-1_ and mobile genetic elements are highlighted in red and yellow, respectively. Regions of > 90% nucleotide sequence identity are marked by grey shading. Δ represents truncated insertion sequences.

We screened the presence of *bla*_CAE-1_ in GenBank database by BLASTn (Accessed by 20 August 2022), and four matches were found, including one plasmid from *C. aquatica* (CP079746, China, water, 2019), and three chromosomes of *P. aeruginosa* (CP042967, patient, Thailand, 2016), *C. aquatica* (LR813086, Spain, Water, 2020) and *Comamonas thiooxydans* (CP063057, China, patient, 2019). Analysis of the adjacent genetic elements of *bla*_CAE-1_ in p1_SCLZS63 and the above four *bla*_CAE-1_-carrying sequences showed that *bla*_CAE-1_ is always reversely located at the immediate upstream of the LysR family transcriptional activator-encoding gene *ampR* (Fig. 2), which consists with the commonly seen *lysR*-accompanied class A β-lactamases as described previously^36^. In all sequences except pB1A, the *bla*_CAE-1_-*ampR* element is bounded by an intact IS*caq2* (upstream of *bla*_CAE-1_) and a ΔIS*caq2* (immediately downstream of *bla*_CAE-1_) that is truncated by a parallel IS*caq1* (Fig. 2). IS*caq1/*IS*caq2* (Accession no. LR813086/CP063057) are IS5 family transposases that are initially identified in *C. aquatica* in the GenBank database*.* However, the flanking IS*caq2* elements are in opposite orientations, which denies the assumption of an IS*caq2*-based composite transposon. In pB1A, the *bla*_CAE-1_-*ampR* element is bracketed by two IS*110* family transposases, which are in the reversed orientation as well (Fig. 2). The exact mechanism of the mobilization of *bla*_CAE-1_ remains unclear.

To investigate the possible origin of *bla*_CAE-1_, BLASTp search of CAE-1 against non-redundant protein database was performed. We found that CAE-1 shows significant amino acid identity with chromosomally encoded class A β-lactamase of bacteria mainly from *Comamonas* and *Acidovorax* sp., which both belong to the family *Comamonadaceae*. Additionally, we found that the average GC-content for the immediate genetic background of the *bla*_CAE-1_ gene (namely, the *bla*_CAE-1_-*ampR* element) is 63.81%, which is close to the chromosomal GC-content of *Comamonas* (64.53% for SCLZS63). These findings suggest that *Comamonadaceae* may be an ancestral source of the *bla*_CAE-1_ gene.

### ***bla***_CAE-1_ and ***bla***_AFM-1_ coexist on a novel type of plasmid p1_SCLZS63

**Figure 3:**
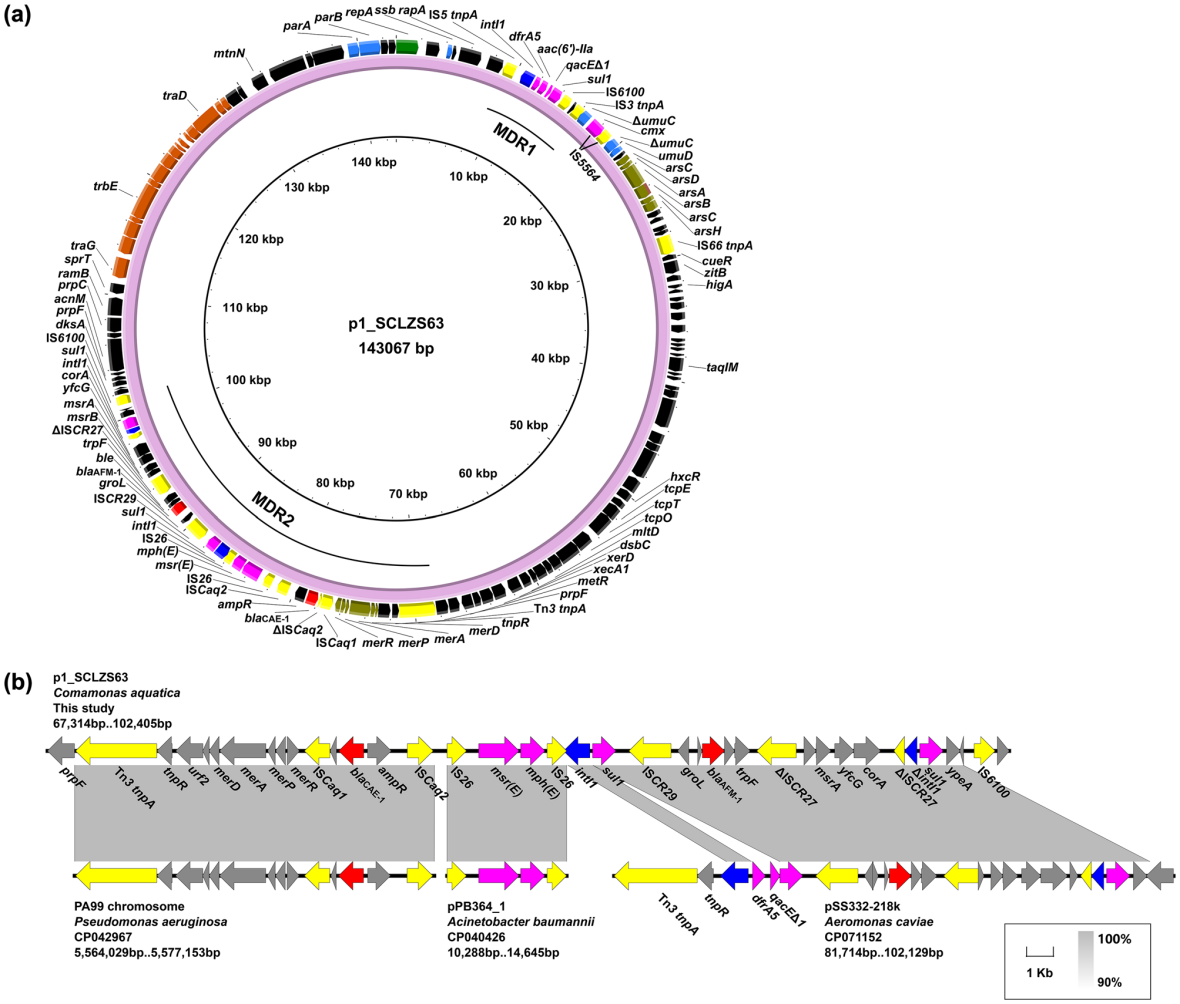
Genetic characterization of p1_SCLZS63. (**a**) Circular organization of p1_SCLZS63. Arrows on the outer ring indicate deduced ORFs and their orientations. Backbone genes for replication, conjugal transfer, and plasmid maintenance are highlighted in green, brown, and light blue, respectively. *bla*_CAE-1_ and *bla*_AFM_ are colored red, and other resistance genes are in fuchsia. Insertion elements, integrase genes, and genes for heavy metal resistance (*ars* and *mer* gene clusters) are indicated in yellow, dark blue, and olive, respectively. Two multidrug-resistant regions (MDR1 and MDR2) are indicated. Δ represents truncated genes. (**b**) Linear comparison of the MDR2 region in p1_SCLZS63 with related regions. Arrows indicate deduced ORFs and their orientations. Regions of > 90% nucleotide sequence identity are indicated by grey shading. The arrow colors are the same as in (**a**).

The resistant plasmid p1_SCLZS63 is 143,067 bp in size, with GC content of 56.68%, and contains 141 ORFs. The backbone of p1_SCLZS63 includes regions responsible for plasmid replication (*repA*), maintenance (*parAB*, *umuCD*), and conjugative transfer (*tra* genes), and it could not be assigned into any known incompatibility group. No similar plasmids were found in the GenBank database using the backbone sequences of p1_SCLZS63 as a query, suggesting that p1_SCLZS63 is a novel type of plasmid. Within the backbone, p1_SCLZS63 harbors two multidrug-resistant (MDR) regions and an arsenate resistance operon (*arsCDABCH*) (Fig. 3A). In the 7.4-kb MDR1 region, the class 1 integron *intI1*-*dfrA5*-*aac(6')-IIa*-*qacEΔ1*-*sul1* is bounded by an IS*5* element (upstream) and an IS*6100* (downstream).

The 32-kb MDR2 region shows a complex structure and is a mosaic with areas of diverse origin (Fig. 3B). The Tn*3*-borne defective mercury resistance operon (*mer*) and the neighboring *bla*_CAE-1_ region were remarkably similar (99.9% identity, 100% coverage) to a region on the chromosome of *P. aeruginosa* strain PA99 (Accession no. CP042967). The *msr*(E)-*mph*(E) unit flanked by IS*26* is universally found in many bacterial families, such as *Enterobacteriaceae* and *Moraxellaceae*. The *bla*_AFM-1_ region resembled that on the plasmid pSS332-218k (Accession no. CP071152) from a clinical *Aeromonas caviae* in Zhejiang, China. In this region, two copies of *intI1* (one complete and one truncated)-*sul1* element in parallel orientation bracketed the core *bla*_AFM-1_ platform, wherein the *bla*_AFM-1_ is located at a conserved fragment IS*CR29*-Δ*groL*-*bla*_AFM-1_-*ble*-Δ*trpF*-ΔIS*CR27*-*msrB-msrA-yfcG-corA*, as had been reported previously in other plasmids^19,20^.

To determine the transfer ability of p1_SCLZS63, conjugation experiments were carried out with *E. coli* J53 as the recipient. Despite repeated attempts, no transconjugants containing p1_SCLZS63 were obtained. In addition, the transfer of p1_SCLZS63 into *E. coli* DH5α by electroporation was also unsuccessful after several attempts.

### Genetic contexts of ***bla***_AFM_ alleles

A total of 14 *bla*_AFM_-bearing plasmids (n = 11) and chromosomes (n = 2) were retrieved from the GenBank database (Accessed on 19 October 2022). *bla*_AFM_ was identified on the chromosomes of *S. maltophilia*, *B. trematum*, and *P. aeruginosa*, and it was also carried by different types of plasmids with various sizes (61,915–495,621 bp) from *Comamonas testosterone*, *A. caviae*, *P. aeruginosa*, *Pseudomonas asiatica*, *A. faecalis*, and *C. aquatica* from China*.* These *bla*_AFM_-bearing plasmids are generally untypable, except that some IncP-2 type plasmids (pAR19640, pNDTH9845, and pWTJH17) carry *bla*_AFM-2_ in *P. aeruginosa*, and an IncW plasmid pAN70-1 carries *bla*_AFM-1_ in *A. faecalis*.

**Figure 4:**
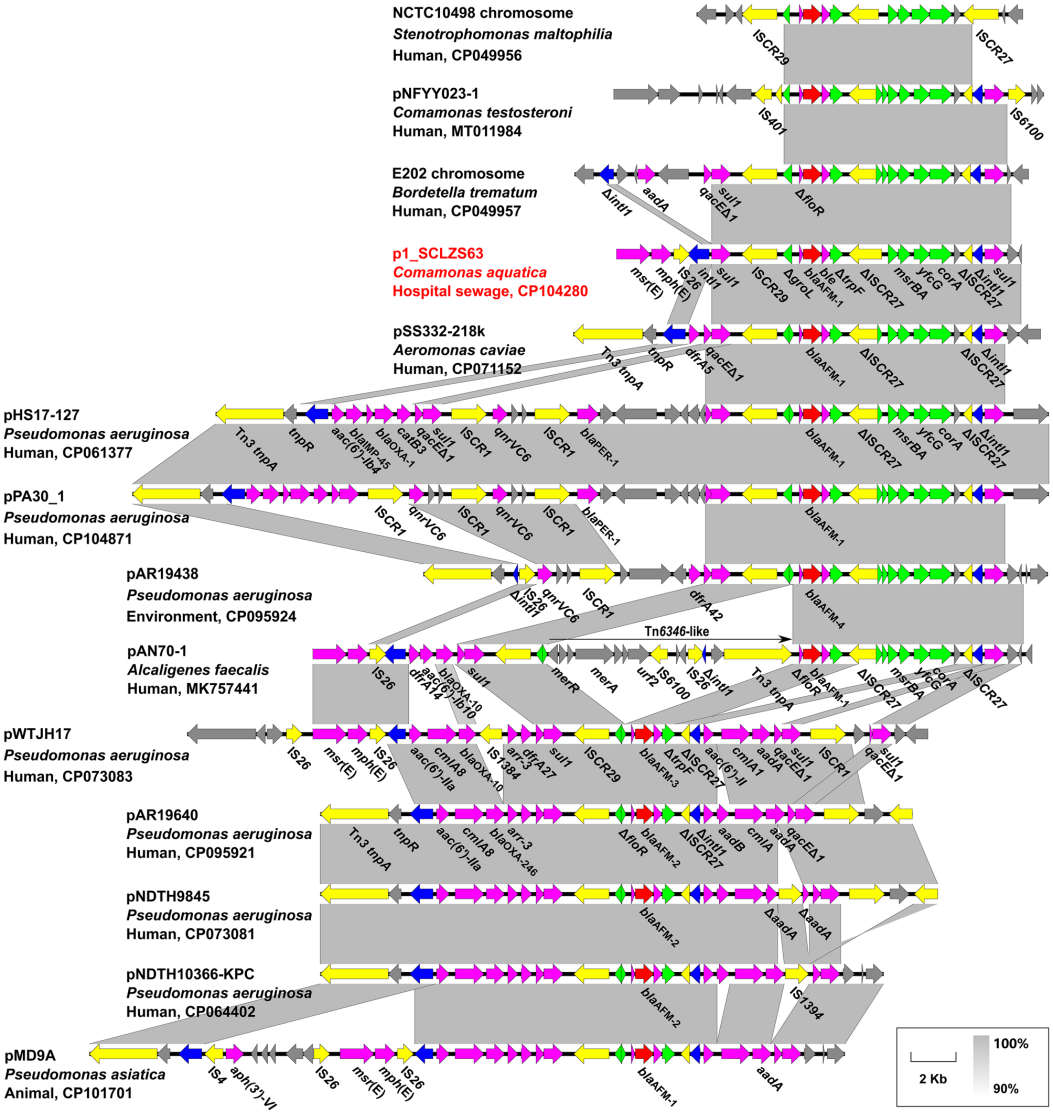
The genetic contexts and mobilization mechanisms of *bla*_AFM_ genes. The position and transcriptional direction of ORFs are indicated by arrows. The *bla*_AFM_ genes are highlighted in red and other resistance genes are in fuchsia. Insertion elements and integrase genes are colored yellow and dark blue, respectively. Genes in the *bla*_AFM_-bearing core module are indicated in green. The strain or plasmid names, species, isolation sources, and GenBank accession numbers are shown. Δ represents truncated genes or elements.

Generally, two kinds of *bla*_AFM_-bearing core modules were identified, namely IS*CR29*-Δ*groL*-Δ*floR*-*bla*_AFM_-*ble*-Δ*trpF*-ΔIS*CR27*-*msrB-msrA-yfcG-corA* (designated type A) and its truncated version IS*CR29*-Δ*groL*-Δ*floR*-*bla*_AFM_-*ble*-Δ*trpF*-ΔIS*CR27* (type B). The truncation of type B seems to have resulted from the recombination event of ΔIS*CR27* (Fig. 4). Almost all the *bla*_AFM-1_ and *bla*_AFM-4_ genes are found in the type A module, except that *bla*_AFM-1_ in pMD9A is in the type B module, in which form *bla*_AFM-2_ and *bla*_AFM-3_ genes are embedded, and that the *bla*_AFM-1_-bearing type A module in pAN70-1 is disrupted by a Tn*6346*-like transposon^19^. In addition, we found that almost all the *bla*_AFM_-bearing core modules are always flanked by class 1 integrons (Fig. 4). The *intI1*-*sul1-bla*_AFM-1_ core module-Δ*intI1*-*sul1* in p1_SCLZS63 in this study seems to be an ancestor structure, from which class 1 integrons with different cassette arrays surrounding the *bla*_AFM_ core module have arisen.

## Discussion

Antimicrobial resistance represents a growing threat to medical care. Infections caused by carbapenem-resistant bacteria are usually associated with poor prognoses and increased morbidity and mortality rates^40^. Close monitoring of carbapenem-resistant bacteria in the hospital sewage is essential. *Comamonas*, especially, carbapenem-resistant *Comamonas*, are abundant in wastewater^16^, while their genome characteristics of antibiotic resistance are poorly characterized. In this work, we isolated a carbapenem-resistant *C. aquatica* from hospital sewage, and its genetic information of drug resistance was characterized by high-resolution WGS. *C. aquatica* has been isolated as the causative agent of bacteremia and septic shock^41^. The prevalence of multidrug-resistance, especially carbapenem resistance in *C. aquatica* warrants further public health surveillance.

Ambler classe A β-lactamases are prevalent and diverse considering other molecular classes, and they represent the most important enzymatic source of both natural and acquired resistance to β-lactams in Gram-negative bacilli^38^. In this study, a new enzyme CAE-1 has been added to the list of class A serine β-lactamase. It exhibits resistance to ampicillin, piperacillin, cefazolin, cefuroxime, and ceftriaxone, as revealed by the in vitro susceptibility analysis. To confirm this, kinetics analysis on pure CAE-1 enzyme would have been informative. With this goal in mind, the *bla*_CAE-1_ gene without its promoter region was cloned in a pET28a expression vector and introduced in an *E. coli* expression strain. However, repeated attempts at expressing and purifying a N-terminus his-tagged version of the CAE-1 enzyme (expected molecular mass of ~ 34 kDa) proved unsuccessful. Possible reasons for why the heterologous expression did not work include low expression under detection limit and an inappropriate host. Further work will be needed to understand the enzyme kinetics of CAE-1.

Plasmids play a vital role in the dissemination of ARGs via horizontal gene transfer. Antibiotic resistance plasmids are rarely reported in *Comamonas*. Two IncP-1 plasmids were previously reported in *Comamonas* from aquatic environments, one of which was associated with the degradation of dyes^42^, and the other one was involved in resistance to heavy metal and oxidative stress^16^. None of the two IncP-1 plasmids carry any ARGs. In the strain SCLZS63, most ARGs are located on the untypable plasmid p1_SCLZS63, including the carbapenem resistance gene *bla*_AFM-1_. The previously reported carbapenemase genes *bla*_GES-5_, and *bla*_IMP-8_ in *Comamonas* are both chromosomally located^16,18^. The emergence of the carbapenemase gene on the plasmid in *Comamonas* has important public health implications, which demands more attention. The conjugation experiments indicated that the *bla*_AFM-1_-harboring p1_SCLZS63 is non-transmissible in this case. While the genomic sequences presented here infer the transfer potential of p1_SCLZS63, the unsuccessful conjugation might result from the exceptionally low transferability that is below detectable limits, or the non-replication of this plasmid due to the unsuitability of *E. coli* J53 recipient strain used in this study for p1_SCLZS63 from *C. aquatica*. Electroporation of p1_SCLZS63 into *E. coli* DH5α was also not successful, which again implies that p1_SCLZS63 may not be readily maintained in a different host of *E. coli* cells.

AFM is a newly identified subclass B1b metallo-β-lactamase, which shows partial identity to the widespread NDM^19^. *bla*_AFM_ has been found in different species of non-fermenting Gram-negative bacteria and is spreading in clinical *P. aeruginosa* isolates at a low rate in China^43,44^. IS*CR* elements are known to move and pick up adjacent genetic components via a rolling-circle mechanism^45^. In the cases of *bla*_AFM_ alleles, IS*CR29* may have initially acquired the Δ*groL*-*bla*_AFM_-*ble*-Δ*trpF*-ΔIS*CR27*-*msrBA-yfcG-corA-*ΔIS*CR27* region and mobilized it upstream of an *intI1*-*sul1* genetic segment, meanwhile truncating the *intI1* gene, followed by a second insertion downstream of the second *intI1*-*sul1* segment. The *intI1*-*sul1-bla*_AFM-1_ core module-Δ*intI1*-*sul1* structure in plasmid like p1_SCLZS63 serves as the intermediate source for the dissemination of *bla*_AFM_, most likely by recombination events. The flanking class 1 integrons have acquired a variety of passenger genes in the subsequent genetic actions, generating complex genetic contexts for *bla*_AFM_.

## Conclusions

In the present study, we identified and characterized a novel class A β-lactamase gene *bla*_CAE-1_ conferring resistance to broad-spectrum cephalosporin, from an environmental *C. aquatica* isolate, where *bla*_CAE-1_ coexists with the carbapenemase gene *bla*_AFM-1_ in a mosaic MDR region on a novel type of plasmid. This finding highlights the importance of the environment as a reservoir of novel antibiotic resistance plasmids and resistance determinants. Effective surveillance is required for understanding the transmission and ongoing evolution of this multidrug-resistance plasmid in clinical settings.

## Data Availability

The complete sequences of the *C. aquatica* SCLZS63 chromosome and plasmids p1_SCLZS63, p2_SCLZS63, and p3_SCLZS63 have been submitted to GenBank with accession numbers CP104279 to CP104282, respectively.
